# Interactions between Benthic Copepods, Bacteria and Diatoms Promote Nitrogen Retention in Intertidal Marine Sediments

**DOI:** 10.1371/journal.pone.0111001

**Published:** 2014-10-31

**Authors:** Willem Stock, Kim Heylen, Koen Sabbe, Anne Willems, Marleen De Troch

**Affiliations:** 1 Department of Biology, Ghent University, Ghent, Belgium; 2 Department of Biochemistry and Microbiology, Ghent University, Ghent, Belgium; University of Otago, New Zealand

## Abstract

The present study aims at evaluating the impact of diatoms and copepods on microbial processes mediating nitrate removal in fine-grained intertidal sediments. More specifically, we studied the interactions between copepods, diatoms and bacteria in relation to their effects on nitrate reduction and denitrification. Microcosms containing defaunated marine sediments were subjected to different treatments: an excess of nitrate, copepods, diatoms (*Navicula* sp.), a combination of copepods and diatoms, and spent medium from copepods. The microcosms were incubated for seven and a half days, after which nutrient concentrations and denitrification potential were measured. Ammonium concentrations were highest in the treatments with copepods or their spent medium, whilst denitrification potential was lowest in these treatments, suggesting that copepods enhance dissimilatory nitrate reduction to ammonium over denitrification. We hypothesize that this is an indirect effect, by providing extra carbon for the bacterial community through the copepods' excretion products, thus changing the C/N ratio in favour of dissimilatory nitrate reduction. Diatoms alone had no effect on the nitrogen fluxes, but they did enhance the effect of copepods, possibly by influencing the quantity and quality of the copepods' excretion products. Our results show that small-scale biological interactions between bacteria, copepods and diatoms can have an important impact on denitrification and hence sediment nitrogen fluxes.

## Introduction

Over the past century anthropogenic activities have dramatically increased the amount of reactive nitrogen on Earth [1]. It has been estimated that nitrogen inputs have increased as much as ten-fold in coastal ecosystems [2], [3]. As a result, these often nitrogen-limited areas [4] have experienced severe eutrophication, resulting in anoxia and changes in community structure [5]. Denitrification and anaerobic ammonium oxidation (anammox) are capable of countering eutrophication by removing reactive nitrogen from the ecosystem as nitrous oxide (N_2_O) or nitrogen gas (N_2_) [6]. In contrast, during dissimilatory nitrate reduction to ammonium (DNRA), nitrate (NO_3_^-^) and nitrite (NO_2_^-^) are reduced to ammonium (NH_4_^+^), preserving reactive nitrogen in the system. In coastal environments, anammox, denitrification and DNRA are all catalysed in the anoxic sediment, but by different microbial assemblages [7].

Denitrification and DNRA are carried out by a different but diverse range of mostly heterotrophic microorganisms [8] and are assumed to be *in situ* mutually exclusive processes determined by the C/N ratio of the system [9]. DNRA is thought to be favoured in nitrate-limited environments rich in labile carbon [9], since the energy yield per nitrate reduced is higher for nitrate ammonification than for denitrification (the reduction of nitrate to ammonia consumes eight electrons rather than five in denitrification, thus more carbon can be oxidised per nitrate reduced; [10]). Anammox is apparently only conducted by members of the *Planctomycetes* group [11] and probably is a less important nitrogen sink than denitrification in nutrient-loaded coastal areas [12].

In the past decades, strong efforts have been made to unravel which benthic organisms affect nitrogen cycling in intertidal sediments and how they do this (e.g. [13], [14]). Macrofauna for example, has been shown to impact DNRA and denitrification by turbating the sediment (e.g. [15], [16]). Other studies focussed on the impact of microphytobenthos (e.g. [17], [18]) on denitrification. The effect of meiofauna (e.g. nematodes and copepods), the intermediate trophic level, on nitrogen fluxes has to date been almost completely neglected [19]. Although the effects of the meiofaunal bioturbation – confined to the superficial sediments – will be far less pronounced than those of macrofaunal bioturbation [20], these organisms can potentially impact benthic nitrate reduction in other ways. Meiofauna is, for instance, capable of eating its body weight equivalent in microorganisms each day [21]. By grazing on microphytobenthos and bacteria, meiofauna will not only counteract the effects of the microphytobenthos and bacteria on nitrate reduction, but also release high amounts of organic nitrogen and carbon [22] into the interstitial environment, thus potentially impacting the C/N ratio. We hypothesized that the meiofauna can impact nitrogen reduction in marine sediments through their grazing activity. The aim of this study was therefore to investigate the impact of meiofauna and its interactions with its food sources, diatoms and bacteria, on denitrification in marine sediments. For this purpose an experiment was setup in which all possible combinations of meiofauna, diatoms and bacteria were included. Harpactecoid copepods were used as meiofauna representative since they occur in high densities at the study site (230±194 ind. 10 cm-^2^, [23]) and have been well-studied in terms of both composition [23] and feeding ecology (e.g. [24]–[26]) in this tidal flat.

In the experiment, both nitrate reduction (the combined activity of denitrification, DNRA and anammox) and denitrification as such were measured as these biochemical reactions are relevant and important ecosystem functions in coastal sediments. Furthermore, nitrate reduction and denitrification can serve as proxies for the overall functioning of the benthic microbial community. In microcosm experiments with sediment, harpacticoid copepods (Crustacea, *Copepoda*) and diatoms from an intertidal flat (Paulina Polder, Westerschelde estuary, The Netherlands), nutrient dynamics and the potential for nitrate reduction and denitrification were monitored. Nitrate reduction and denitrification rates could not be measured *in situ*, as they are largely anaerobic processes whilst copepods are strictly aerobic. Both rates were therefore measured indirectly by making a subsample of the homogenised microcosm anaerobic and measuring the potential rates under non-carbon or -nitrogen limiting conditions.

## Methodology

### Field sampling

Silty sediment was collected from the intertidal mudflat Paulina (Westerschelde estuary, The Netherlands; 51°20′ N, 3°43′ E) in February 2013 by scraping the top layer (0–3 cm) of the sediment at low tide. Seawater (salinity: 19.3; 1.85±1.11 µM NO_2_; 122.02±8.17 µM NO_3_^-^; 2.50±1.70 µM NH_4_^+^; 0.73±0.60 µM PO_4_^3-^; 88.89±0.16 µM Si^4+^; N = 3) was collected from the same site and was filtered over a 0.22 µm filter (Corning 500 mL Bottle Top Vacuum Filter) and stored in the dark at 4°C (filtered seawater: FSW). No permits were required for the sampling nor were there any endangered or protected species involved.

### Experimental setup

Collected sediment was washed over a 250 µm sieve to remove all benthic fauna. The sieved sediment (average median grain size: 56.89±0.25 µm; N = 3) was divided in equal aliquots of 80 g in polyethylene containers (microcosms). The microcosms with ±2.5 cm of sieved sediment were stored frozen (−20°C) to kill all the remaining fauna. A microcosm was defrosted two days before the start of a treatment. After adding 60 ml of filtered seawater (FSW), the thawed sediment was thoroughly mixed. Right before the start of a treatment, the FSW was drained off.

The experimental design included a blank: the defaunated sediment in which only bacteria were present. To verify the effect of copepods and diatoms, independently of one another, on the bacteria, they were added separately to the microcosm. To cover the interaction effects between copepods and diatoms, both of them were added to the microcosm. In order to discriminate between the effects of the activity of the copepods themselves and the waste products that they produce, a treatment was included in which the spent medium from copepods was added to the microcosm.

Together with the positive control (increased NO_3_^-^), this resulted in a total of six different treatments.

Each treatment starting with a microcosm containing 80 g of defaunated and thawed sediment: (1) Blank: +70 ml FSW; (2) Increased NO_3_^-^: +0.1 mmol KNO_3_ in 70 ml FSW; (3) Copepod: +200 phototactic copepods collected from Paulina Polder in 70 ml FSW; (4) Diatom: +4×10^5^ cells of *Navicula* sp. (36.27±2.30 µm; isolated from the study site in 2012) in 70 ml FSW (corresponds to chlorophyll a concentrations observed in the study site.); (5) Copepod+diatom: +4×10^5^ cells of *Navicula* sp. and 200 copepods (as above) in 70 ml FSW; (6) Spent medium:+70 ml of FSW, in which 200 copepods were fed 4×10^5^ *Navicula* cells over a period of one week. A visual (microscopic) screening of the treatments revealed that most diatoms had been eaten by the copepods after one week. Prior to the start of the treatment this spent medium was stored at −20°C after manually removing the copepods.

One hour after the start of the experiment, 10 ml of water was extracted for nutrient analysis (initial nutrient concentration) from each microcosm. All microcosms were then incubated for 7.5 days, at 15°C.

To study the effects of the photosynthetic activity of the diatoms on the nitrogen fluxes, all treatments were run (1) under a diurnal (12 h/12 h) light regime and (2) in the dark. Cold-white fluorescent lamps provided the necessary light at a rate of 20–25 µmol photons m^−2^ s^−1^. Four replicates were used for each treatment under each light condition. To avoid depletion of active nitrogen in the microcosms, half of the SW was renewed on day 5 and 6 of the experiment. At the end of the incubation period, 10 ml of SW was stored for nutrient analysis (final nutrient concentration).

An additional experiment was setup in which the blank and copepod+diatom treatment were repeated to verify the effects of copepods and diatoms on the oxygen pentration depths (Text S1).

Denitrification rates were measured using the so-called acetylene inhibition method [27] [16] (cf. Fig S1 which illustrates the design of the experiment). In the presence of acetylene the final reaction of denitrification, in which N_2_O is converted to N_2_, is inhibited, causing N_2_O to accumulate. The easily quantifiable N_2_O can then be measured as a proxy for denitrification. The rate at which NO_3_^-^ is consumed is a good proxy for the combined activity of all three reduction pathways.

At the end of each treatment, a serum vial was filled with 30 g (wet weight) sediment and 20 ml incubation water (collected from the treatments; Fig S1). To prevent nitrogen and carbon limitation, the water was supplemented with 0.5 mmol KNO_3_ and 1 mmol α-D-glucose. After vigorous shaking, 1 ml was extracted to determine the initial NO_3_^-^/NO_2_^-^ concentration (t_0_). The vials were hermetically sealed and flushed five times with helium to remove oxygen. After adding 10% acetylene, the vials were incubated at 25°C under a constant stirring rate of 90 rpm. The N_2_O concentrations were measured every two hours by injecting 1 ml of headspace in a GC-TCD (Gas Chromatography- Thermal Conductivity Detector; MICRO E-0391, Interscience; LOD 13.55 ppm N_2_O). This was done at four time points (t_1_–t_4_) for each serum vial (Fig. S1). N_2_O concentrations were corrected for headspace volume changes, pressure and dissolving of the gas into the liquid phase. To ascertain potential side-effects of acetylene [28], the process was repeated with a technical replicate without acetylene (data not shown). At the second (t_2_) and final sampling event (t_4_) from the replicate without acetylene, 800 µl of fluid was extracted for later NO_3_^-^/NO_2_^-^ determination (indicative for the NO_3_^-^ reduction activity; Fig. S1).

### Nutrient analysis

Nutrient concentrations (NO_3_^-^, NO_2_^-^, NH_4_^+^ and PO_4_^-^) of the samples collected at the start and the end of the incubation period (initial and final nutrient concentration) were analysed with an automatic chain (SAN ^plus^ segmented flow analyser, SKALAR) according to Beyst et al. [29].

Samples extracted from the serum vials for NO_3_^-^/NO_2_^-^ determination were analysed differently because the above used method required higher sample volumes. The samples were centrifuged (14000 rpm, 5 min) and the supernatants were stored frozen (−20°C) prior to analysis. Analysis of NO_3_^-^ and NO_2_^-^ was based on a colorimetric method as described by Cataldo et al. [30], based on Griess [31] with adjustments from Navarro-Gonzálvez et al. [32].

### Data analysis

The software package R 2.15.0. was used for data analysis. Differences in initial and final nutrient concentrations between the treatments and light conditions were detected using a two-way ANOVA on the rank transformed concentrations, performed in the software package R 2.15.0. Pairwise differences were unravelled using Dunnett's Modified Tukey-Kramer Pairwise Multiple Comparison Test (DTK) [33] using 95% confidence limits.

N_2_O production rates and NO_3_^-^ reduction rates were calculated according to both Magalhaes et al. [34], assuming no bacterial growth between the N_2_O, respectively NO_3_^-^/NO_2_^-^, samplings (i.e. between t_0_ and t_4_), and Stenström et al. [35], assuming that bacterial growth does occur. Magalhaes et al. [34] obtained the N_2_O production rates by dividing the N_2_O concentration at t_4_ by t_4_. Stenström et al. [35], however, propose an exponential regression to accommodate for the increasing gas production rate between samplings: 
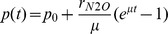
, with p =  the amount of gas at time t, p_0_ =  the amount of product at t_0_, r_N2O_  =  the N_2_O production rate (see below) and µ =  the specific growth rate constant. Since the serum vials were flushed with helium, there was no N_2_O at the start of the incubation and p_0_ was set to zero. This function was adapted to fit the data for the NO_3_^-^ reduction (in the serum vials) to 
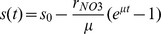
, with s =  the amount of substrate (NO_3_^-^) at time t, s_0_ =  the amount of substrate at t_0_ and r_NO3_ =  the initial NO_3_^-^ reduction rate, further referred to as “NO_3_^-^ reduction rate”.

The N_2_O production rates (r_N2O_) obtained from the regression analysis are the production rates of N_2_O at the start (t_0_) of the denitrification potential experiment, i.e. after the 7.5 days incubation period (Fig. S1). Likewise, the initial NO_3_^-^ reduction rates (r_NO3_) express the NO_3_^-^ reduction rate at the start of the denitrification potential experiment. Thus the rates are corrected for any microbial growth occurring after the NO_3_^-^ and glucose addition.

Differences in the obtained N_2_O production and the NO_3_^-^ reduction rates between the different treatments and light conditions were analysed using a permutation-based two-way ANOVA [36] since the data were not normally distributed. Pairwise differences were analyzed using Wilcoxon rank-sum post-hoc test using 95% confidence limits, with Bonferroni correction.

## Results

### Viability

At the end of the incubation, viability of copepods and diatoms were checked in all treatments. Both microscopic observations (after collecting the cells with the lens-tissue method) and pulse-amplitude modulation (PAM; Maxi-Imaging PAM M-series, Walz) showed healthy and active diatom cells. Visual observations showed active copepods in all microcosms of the copepod and copepod + diatom treatment.

### Nutrient levels

#### Initial nutrient concentrations

Except for the spent medium and the increased NO_3_^-^ treatment, the initial nutrient concentrations in the microcosms did not differ among treatments (ANOVA; p<0.05; Table 1). The nitrate concentration in the spent medium was significantly lower than in the other treatments, while in the increased NO_3_^-^ treatment, the nitrate concentration was, as expected, significantly higher. Nitrite was significantly lower in the spent medium and in the increased NO_3_^-^ treatment. The initial ammonium concentration was highest in the copepod + diatom treatment, but the difference was only significant compared to the increased NO_3_^-^ treatment. In contrast, the phosphate concentration in the spent medium treatment was 2.5–4 times higher than in the other treatments.

**Table 1:** Initial and final nutrient concentrations of the different treatments.

Nutrients	NO_3_^-^, mean ±SE (µM)		NO_2_^-^, mean ±SE (µM)		NH_4_^+^, mean ±SE (µM)		PO_4_^3-^, mean ±SE (µM)	
**Treatments**	Initial***	Final	Initial***	Final	Initial*	Final**	Initial***	Final***
**Blank**	125.93±5.00^a^	2.24±0.79	4.35±0.20^a^	0.23±0.10	99.67±6.54^ab^	360.03±24.42^a^	0.85±0.13^a^	18.90±1.90^ ab^
**Increased NO_3_^-^**	1135.29±258.98^b^	1.29±0.36	3.43±0.21^b^	0.25±0.03	105.56±5.06^a^	400.71±22.01^ ab^	0.75±0.11^a^	15.30±1.74^a^
**Copepod**	424.17±265.21^a^	0.83±0.24	4.48±0.21^a^	0.32±0.06	89.13±6.27^ ab^	467.19±9.70^b^	0.98±0.16^a^	23.27±2.36^b^
**Diatom**	130.86±4.04^a^	0.76±0.21	4.17±0.14^a^	0.20±0.03	88.97±6.75^ ab^	372.18±21.35^a^	0.94±0.17^a^	13.79±1.83^a^
**Copepod+diatom**	131.17±2.95^a^	1.43±0.58	4.39±0.27^a^	0.65±0.18	78.16±2.25^b^	485.69±34.52^ ab^	0.66±0.09^a^	25.98±2.61^b^
**Spent medium**	34.33±10.63^c^	0.65±0.31	1.42±0.32^c^	0.17±0.05	94.43±6.97^ ab^	425.93±16.24^ ab^	2.44±0.30^b^	26.29±2.53^b^

Significant differences of the ANOVAs indicated with symbols (*** ≤0.001<** ≤0.01<* ≤0.05< ˙ <0.1). The different superscripted letters indicate significant differences (P<0.05; DTK) between the treatments. Light conditions (not shown) did not alter the outcome of treatments (see text).

#### Final nutrient concentrations

After 7.5 days of incubation, the nutrient concentrations did not differ significantly between light conditions (two-way ANOVA; p>0.05).

At the end of the incubation period, nitrate and nitrate were almost completely depleted in all treatments (on average 1.12±0.03 µM and 0.30±0.01 µM, respectively; Table 1), despite renewal of half of the SW on days 5 and 6. The ammonium concentration more or less quadrupled towards the end of the experiment (average 384.08±3.04 µM). The final ammonium concentration in the copepod treatment was significantly higher than in the diatom and the blank treatment. The final ammonium concentration in the copepod + diatom treatment was the highest of all treatments, although it was not significantly different from the other treatments due to considerable variation between the replicates.

The same pattern was observed in the final phosphate concentrations. The phosphate concentrations strongly increased during the incubation period as the final average phosphate concentration (18.60±0.21 µM) was almost twenty times higher than the initial one.

### Potential for nitrate reduction and denitrification

During the measurement of the denitrification potential, nitrate was consumed, while nitrite and nitrous oxide were produced (see Fig. S2 for the average NO_3_^-^, NO_2_^-^ and N_2_O concentrations during the measurement of the denitrification potential). Since the exponential function proposed by Stenström et al. [35] had a significantly better fit than the linear regression proposed by Magalhaes et al. [34], rates were calculated with the exponential function.

**Figure 1:**
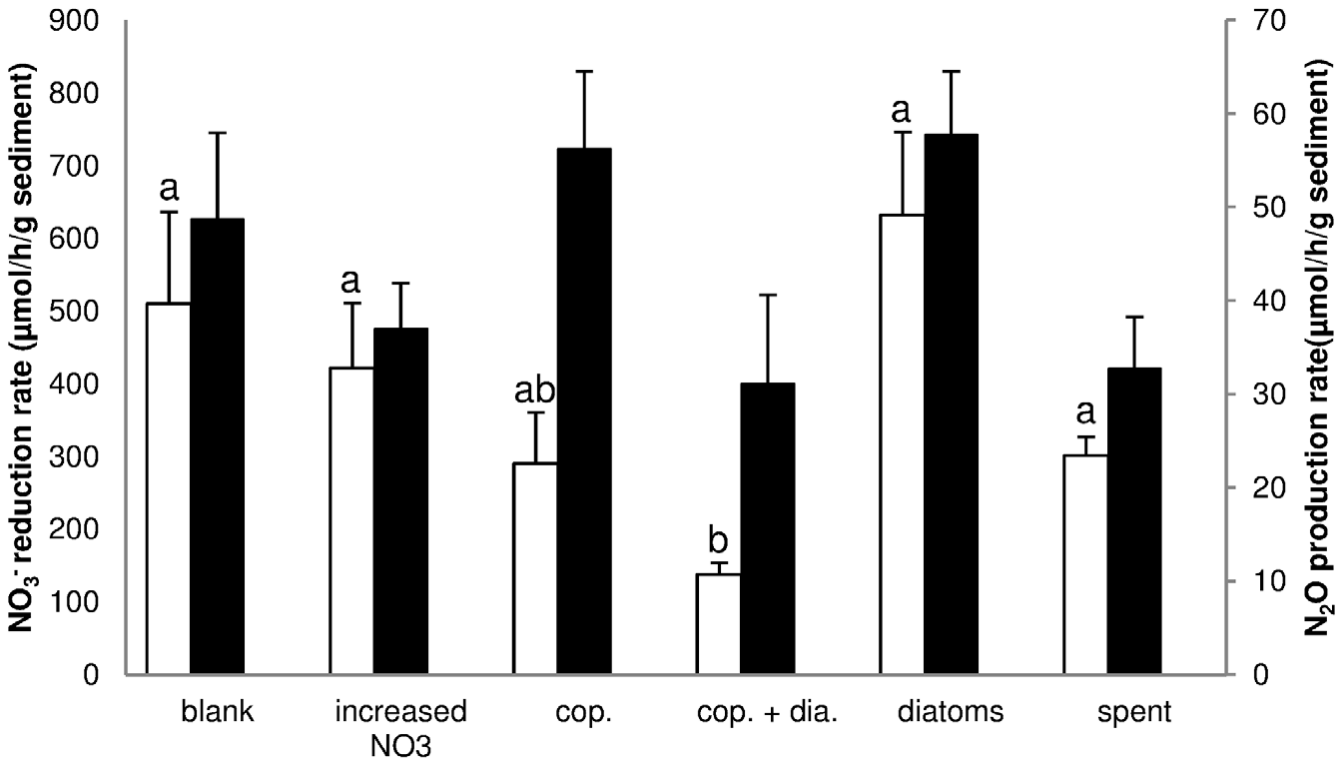
NO_3_^-^ reduction and N_2_O production rates. NO_3_^-^ reduction rate (black bars; left y-axis) and N_2_O production rates (white bars; right y-axis) during the measurement of the denitrification potential for the different treatments (mean ± SE; cop.  =  copepods; cop. + dia.  =  copepod+ diatom). The different letters above the bar indicate significant differences (P<0.05; DTK) between the treatments. Light conditions (not shown) did not affect the outcome of treatments.

The NO_3_^-^ reduction and N_2_O production rates did not differ between light conditions. In contrast to the NO_3_^-^ reduction rate, which did not differ between treatments (Fig 1, black bars), the N_2_O production rate did significantly differ between the treatments (p<0.01; Fig. 1, white bars). The N_2_O production rate was significantly lower for the copepod + diatom treatment compared to the blank (p<0.001), diatom (p<0.001), increased NO_3_^-^ (p<0.001) and spent medium (p<0.001) treatments.

The correlation between N_2_O production and NO_3_^-^ reduction rates was weak but significantly positive (Pearson's r = 0.30, p<0.05).

## Discussion

### Effect of copepods

The presence of copepods or their spent medium resulted in elevated phosphate and ammonium concentrations in the microcosms. The copepods' outfluxes (including excretion products, moults, remnants of sloppy feeding) therefore proved to be an important source of both N and P which are, in coastal areas, potentially limiting nutrients [37].

**Figure 2:**
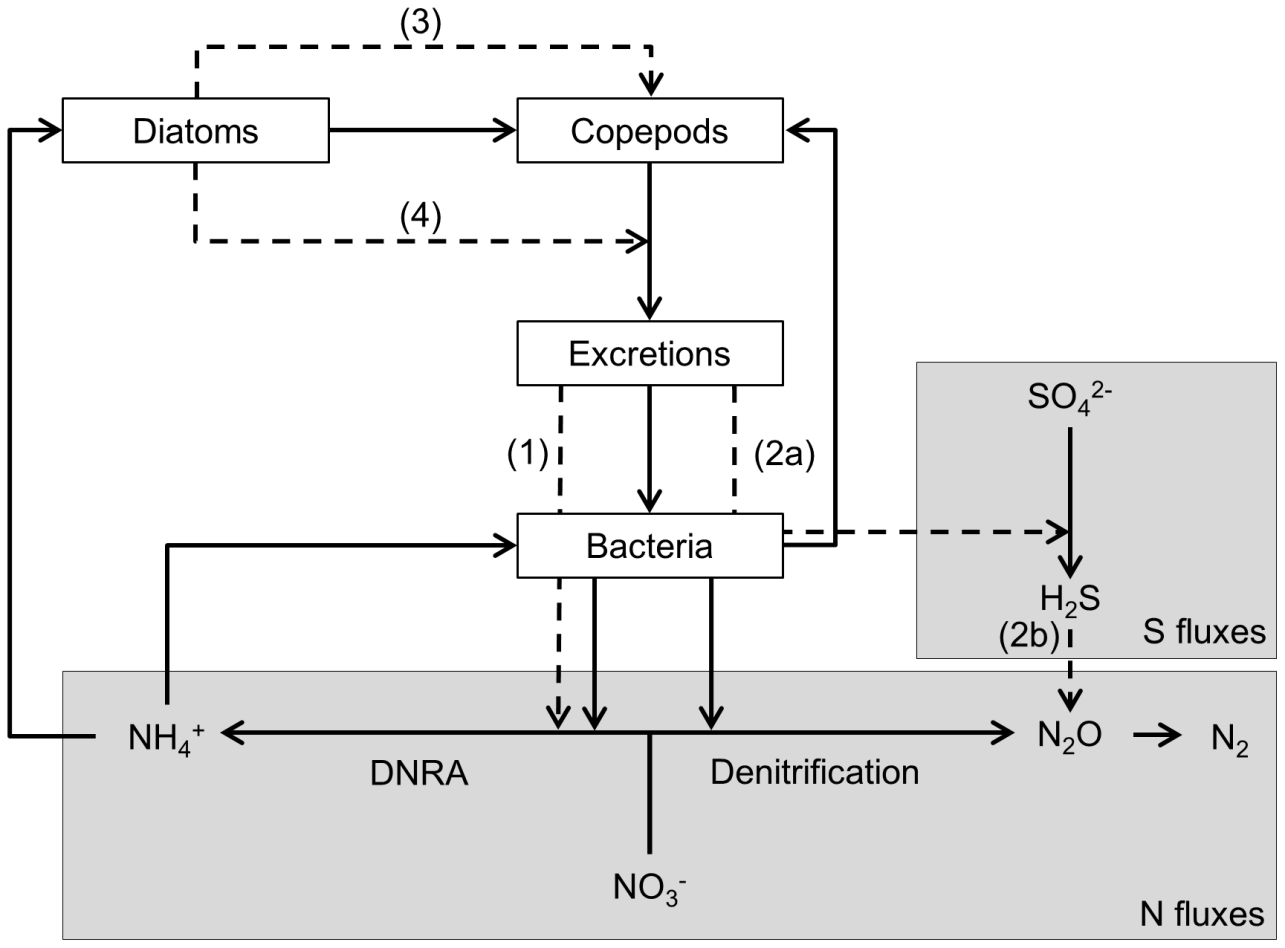
Summary of the assumed interactions to explain the observed differences in N_2_O production rates. The assumed interactions which affect denitrification are indicated with dashed arrows. Bacteria mediated relevant reduction reactions of the nitrogen pathway and sulfur pathway are enclosed by grey boxes indicated with respectively ‘N fluxes’ and ‘S fluxes’. Copepods feed on both diatoms and bacteria, and produce excretion producs (excretions). Bacteria feed on the excretion produces and are also responsible for the reduction of SO_4_^2-^ to H_2_S and of NO_3_^-^ to NH_4_^+^ (DNRA) and N_2_O+N_2_ (denitrification) in the microcosm. The produced NH_4_^+^ is assimilated by both bacteria and diatoms. Copepods affect the N_2_O production rate by producing excretion products which provide an extra carbon source, of which mainly the DNRA bacteria can take advantage (1) and also enhances SO_4_^2-^ reduction (2a), which results in more H_2_S. The increased H_2_S inhibits denitrification (2b). Diatoms have no direct effect on the N_2_O production rate, but do have an indirect effect by enhancing the survival of the copepods (3) and influencing the quantity and composition of the copepods' excretion products (4).

Contrary to macrofauna, not much is known about the impact of meiofauna on nitrogen fluxes. Macrofauna seems to have its biggest impact through bioturbation (e.g. [15]). However, the oxygen penetration depth did not increase in the presence of copepods (Text S1), and consequently the effects of copepods are not related to oxygen. Since the N_2_O production rates of the copepod and their spent medium treatments were low, the copepods appeared to negatively impact denitrification, at least partially via their outfluxes. The outfluxes of the copepods are, apart from being an important nutrient source, a source of organic compounds and hence a substrate for bacterial growth [38]. Since organic matter loading may be one of the most important variables controlling denitrification in aquatic ecosystems [39], the impact of these carbon outfluxes should not be underestimated. The copepod outfluxes contain high amounts of labile carbon [22], which are known to stimulate DNRA over denitrification and anammox (Fig. 2, dashed arrow 1; [9]). Copepods can also indirectly stimulate labile carbon production by mechanically breaking down detrital particles [40], [41]. In addition, more organic matter results in a higher sulphate reduction rate (Fig. 2, dashed arrow 2; [42]). The main product of sulphate reduction is hydrogen sulphide, which inhibits denitrification and nitrification, but not DNRA [7]. These findings are supported by the higher final ammonium concentrations in the treatments with copepods or their spent medium. Furthermore, the NO_3_^-^ reduction rate did not differ significantly between the treatments, suggesting that the reduced denitrification activity in the treatments with copepods or their spent medium was compensated by another nitrate reducing process.

### Effects of diatoms

Since there were no differences between the final nitrate, nitrite and ammonium concentrations of the blank and the diatom treatment, diatoms seemed to have no net effect on the nitrogen fluxes in the microcosm. Likewise, N_2_O production rates did not differ between the blank and the diatom treatment either. It thus appears that diatoms had no or very little impact on denitrification in the microcosms. This is inconsistent with previous reports, where the presence of benthic microalgae generally had a negative impact on denitrification rates [43] as microalgae outcompeted the bacteria for nitrogen [18]. The opposite has however also been observed (e.g. [44]) were microalgae enhanced denitrification. It is unlikely that the inoculation concentration (2×10^5^ diatoms/cm^^2^^) used in this study was too low to have a significant effect on the denitrification rate as it was comparable to the diatom concentrations in other studies were diatoms did have an effect on the nitrogen fluxes (e.g. [18]). It is, however, possible that positive and negative effects of the diatoms cancelled each other out or that the overall effect was too small to be detected. However, one should bear in mind that the used incubation time might be too short to obtain strong effects caused by the diatoms [18], [45].

### Effects of diatoms + copepods

The negative effect of the copepods on denitrification was most pronounced and only significant when diatoms were added to the system. This suggests an important interaction effect between diatoms and copepods. Since the diatoms themselves had no effect on denitrification and nitrate reduction, it is unlikely that they were directly responsible for the difference between the copepods + diatoms and the copepods treatments. The diatoms can, however, influence the composition of the copepod outfluxes (Fig. 2, dashed arrow 4), which depends on the food type [22]. As diatoms are thought to be a better food source for copepods than bacteria (e.g. [26], [46]), they might also enhance the survival of the copepods and, accordingly, their activity and the quantity of the excretion products (Fig. 2, dashed arrows 3–4).

### Effects of the nitrate addition

The N_2_O production rate for the increased NO_3_^-^ treatment was unexpectedly similar to the blank. In general, the denitrification rate is positively influenced by the nitrate load (e.g. [47], [48]), but such a relation was not observed here. However, a preliminary test in which we used a shorter, three and a half day long incubation period showed that the N_2_O production rate was almost twice as high for the increased NO_3_^-^ treatment compared to the blank (data not shown). This indicates that the supplemented nitrate was depleted within the first few days and that the denitrifying community changed accordingly.

### Final considerations

Our findings have potentially important implications for our understanding of nutrient fluxes in marine sediment and the role of meiofauna. It was already known that meiofauna facilitates biomineralization of organic material and enhances nutrient regeneration [40]. This is an indirect process, by stimulating the bacterial community [40] through the production of excretion products [49] and bioturbation [40]. The present study suggests that these processes may also negatively impact denitrification. Consequently, more active nitrogen will be preserved in the ecosystem in the presence of meiofauna. Furthermore, they also increase the freely available phosphorus and carbon (this study, [22]). These elevated nutrient levels will benefit both bacteria and microphytobenthos. Our observations should, however, be interpreted with care as they were obtained from a short-term microcosm experiment. They might therefore not be representative for the highly dynamic estuarine sediments these organisms where obtained from. Our results do, however, prove that the interactions between meiofauna, diatoms and bacteria can potentially impact the nitrogen fluxes and that they should therefore not be neglected in future research. The time-dependent effect of the increased NO_3_^-^ treatment clearly illustrates the importance of the temporal scale in this setup. It might therefore prove useful for further research to investigate the effects of the different treatments at different time intervals. Furthermore, additional experiments (for instance relying on the ^15^N technique; [50]) will be necessary to fully unravel these fine-scale interactions.

## Supporting Information

Figure S1 **Design of the denitrification potential experiment.** Incubation steps are indicated by dotted arrows. Manipulations are indicated by a dashed arrow. Each microcosm (here represented as a rectangle) was incubated for 7.5 days, after which water (blue) and sediment (brown) were transferred from the microcosm to the serum vials (represented as trapezoids). Glucose and potassium nitrate were added to the vial after which it was thoroughly homogenized. The headspace was flushed with helium to remove oxygen, after which acetylene was injected. The vials were sampled four times for N_2_O determination, every two hours.
(DOC)

Figure S2 **NO3-, NO2- and N2O concentrations in the serum vials after the addition of 0.5 mmol KNO3 during the measurement of the denitrification potential (from t0 to t4).** Amounts (µmol/h/g sediment) are averaged over all samples (all treatments), as is the time at with the sample was taken (Time; h:min) ± SE. N2O concentration(blue) plotted on the right y axis; NO3- (red) and NO2- (green) concentrations on the left y axis.
(DOC)

Text S1 **Measurement of oxygen penetration depth.** To verify the effects of copepods and diatoms on the oxygen pentration depths, the experiment as described in the method section was repeated for the blank, diatom and copepod+diatom treatment. The methodology and results of the additional experiment are shown here.
(DOCX)
