# Supplementary material for: Interactions between Benthic Copepods, Bacteria and Diatoms Promote Nitrogen Retention in Intertidal Marine Sediments
Source: PLoS One. 2014 Oct 31;9(10):e111001. doi: 10.1371/journal.pone.0111001 (PMC4215923; doi:10.1371/journal.pone.0111001)
Supplement: Figure S1 — Design of the denitrification potential experiment. Incubation steps are indicated by dotted arrows. Manipulations are indicated by a dashed arrow. Each microcosm (here represented as a rectangle) was incubated for 7.5 days, after which water (blue) and sediment (brown) were transferred from the microcosm to the serum vials (represented as trapezoids). Glucose and potassium nitrate were added to the vial after which it was thoroughly homogenized. The headspace was flushed with helium to remove oxygen, after which acetylene was injected. The vials were sampled four times for N2O determination, every two hours. (DOC) [file pone.0111001.s001.doc]

**Figure S1. Design of the denitrification potential experiment.**


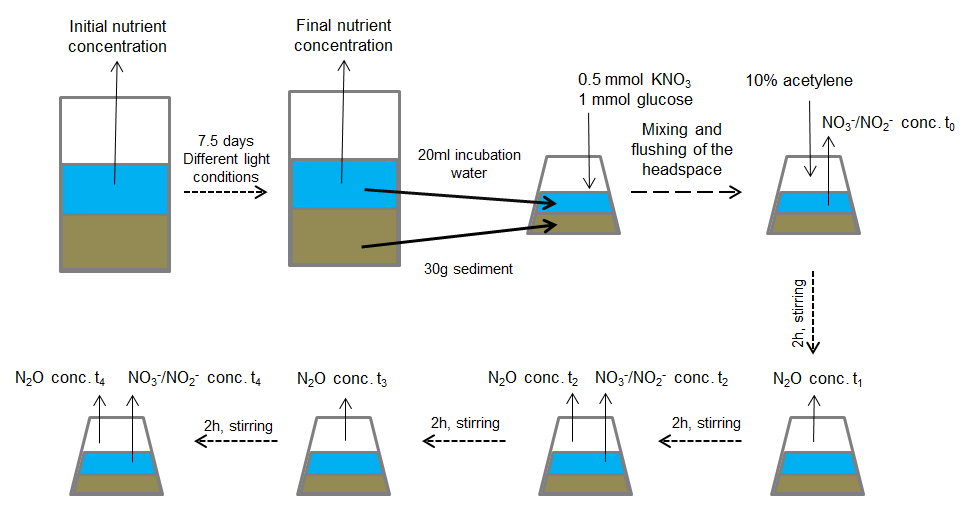


Incubation steps are indicated by dotted arrows. Manipulations are indicated by a dashed arrow. Each microcosm (here represented as a rectangle) was incubated for 7.5 days, after which water (blue) and sediment (brown) were transferred from the microcosm to the serum vials (represented as trapezoids). Glucose and potassium nitrate were added to the vial after which it was thoroughly homogenized. The headspace was flushed with helium to remove oxygen, after which acetylene was injected. The vials were sampled four times for N_2_O determination, every two hours.
