# Supplementary material for: Interactions between Benthic Copepods, Bacteria and Diatoms Promote Nitrogen Retention in Intertidal Marine Sediments
Source: PLoS One. 2014 Oct 31;9(10):e111001. doi: 10.1371/journal.pone.0111001 (PMC4215923; doi:10.1371/journal.pone.0111001)
Supplement: Figure S2 — NO3-, NO2- and N2O concentrations in the serum vials after the addition of 0.5 mmol KNO3 during the measurement of the denitrification potential (from t0 to t4). Amounts (µmol/h/g sediment) are averaged over all samples (all treatments), as is the time at with the sample was taken (Time; h:min) ± SE. N2O concentration(blue) plotted on the right y axis; NO3- (red) and NO2- (green) concentrations on the left y axis. (DOC) [file pone.0111001.s002.doc]

**Figure S2. NO_3_^-^, NO_2_^-^ and N_2_O concentrations in the serum vials after the addition of 0.5 mmol KNO_3_ during the measurement of the denitrification potential (from t_0_ to t_4_).**


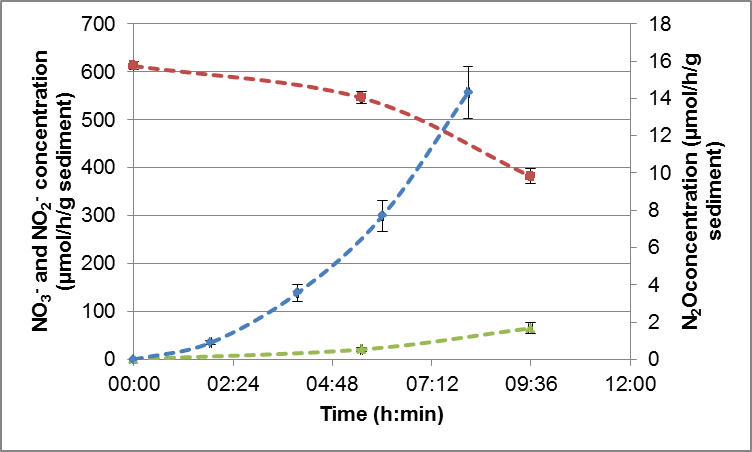


Amounts (µmol/h/g sediment) are averaged over all samples (all treatments), as is the time at with the sample was taken (Time; h:min) ± SE. N_2_O concentration(blue) plotted on the right y axis; NO_3_^-^ (red) and NO_2_^-^ (green) concentrations on the left y axis.
