# Supplementary material for: Interactions between Benthic Copepods, Bacteria and Diatoms Promote Nitrogen Retention in Intertidal Marine Sediments
Source: PLoS One. 2014 Oct 31;9(10):e111001. doi: 10.1371/journal.pone.0111001 (PMC4215923; doi:10.1371/journal.pone.0111001)
Supplement: Text S1 — Measurement of oxygen penetration depth. To verify the effects of copepods and diatoms on the oxygen pentration depths, the experiment as described in the method section was repeated for the blank, diatom and copepod+diatom treatment. The methodology and results of the additional experiment are shown here. (DOCX) [file pone.0111001.s003.docx]

**Supplementary text S1: Measurement of oxygen penetration depth**

*To verify the effects of copepods and diatoms on the oxygen pentration depths, the experiment as described in the method section was repeated for the blank, diatom and copepod+diatom treatment.*

**Methodology**

Three microcosms (replicates) of the blank, diatom and copepod+diatom treatment were setup as described in the Method section of Stock et al. (2014). The 9 microcosms were incubated under a diurnal (12h/12h) light regime (cf. Method section). After 7.5 days, the oxygen profiles were measured using a Unisense oxygen microsensor (type ox 100). Profiles were recorded in vertical increments of 250 µm. The average oxygen penetration depth was calculated from three consecutive profiles of each of the microcosms. The oxygen penetration depth was compared between the treatments by means of one-way ANOVA in R 3.0.2.

**Results**

Oxygen penetration depth did not differ significantly between treatments (p>0.05; Fig. S1-1).

**Figure S1-1. Oxygen penetration depth in the microcosms after an incubation of 7.5 days.** (Average +SE)
